# Achieving view-distance and -angle invariance in motion prediction using a simple network

**DOI:** 10.1186/s42492-024-00176-5

**Published:** 2024-10-28

**Authors:** Haichuan Zhao, Xudong Ru, Peng Du, Shaolong Liu, Na Liu, Xingce Wang, Zhongke Wu

**Affiliations:** 1 School of Artificial Intelligence, Beijing Normal University, Beijing, 100875 China; 2 School of Arts and Communication, Beijing Normal University, Beijing, 100875 China; 3 Information Science Academy of China Electronics Technology Group Corporation, Beijing, 10587 China

**Keywords:** Geometric coding, Motion prediction, Motion space, View distance invariance, View angle invariance, Multi-layer perceptrons

## Abstract

Recently, human motion prediction has gained significant attention and achieved notable success. However, current methods primarily rely on training and testing with ideal datasets, overlooking the impact of variations in the viewing distance and viewing angle, which are commonly encountered in practical scenarios. In this study, we address the issue of model invariance by ensuring robust performance despite variations in view distances and angles. To achieve this, we employed Riemannian geometry methods to constrain the learning process of neural networks, enabling the prediction of invariances using a simple network. Furthermore, this enhances the application of motion prediction in various scenarios. Our framework uses Riemannian geometry to encode motion into a novel motion space to achieve prediction with an invariant viewing distance and angle using a simple network. Specifically, the specified path transport square-root velocity function is proposed to aid in removing the view-angle equivalence class and encode motion sequences into a flattened space. Motion coding by the geometry method linearizes the optimization problem in a non-flattened space and effectively extracts motion information, allowing the proposed method to achieve competitive performance using a simple network. Experimental results on Human 3.6M and CMU MoCap demonstrate that the proposed framework has competitive performance and invariance to the viewing distance and viewing angle.

## Introduction

**Fig. 1:**
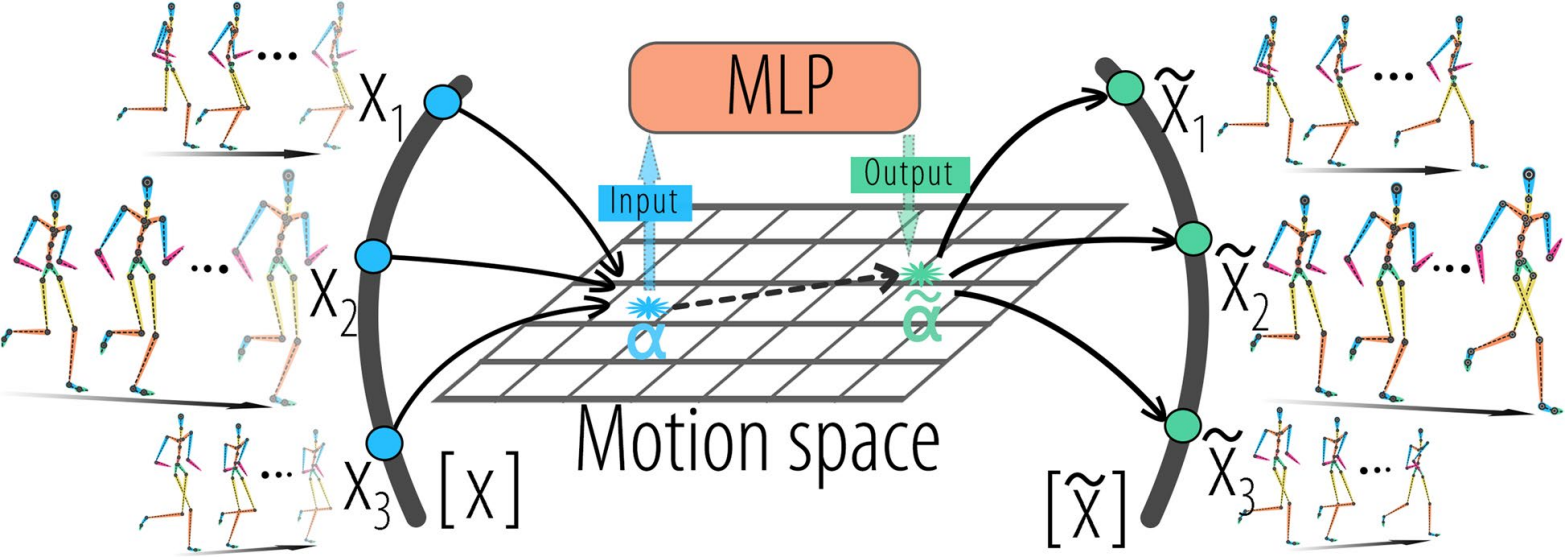
Illustration of the proposed method. [*X*] is the equivalence class of $X_1, X_2$ and $X_3$. Motions are first encoded into a flattened motion space and then motion prediction is accomplished by multi-layer perceptrons (MLPs) based network

Human motion prediction has garnered significant attention for its successful application in various domains, including autonomous driving [1, 2], human behavioral understanding [3, 4], and multimedia [5, 6]. Data-driven methods have led to significant breakthroughs in human motion modeling [7, 8]. However, the models are effective under the strong assumption that all action samples have consistent viewing distances and angles, which may not hold in practical scenarios. Figure 1 illustrates the variability in the viewing distance and angles, where the viewing distance can vary, and the captured motions may be from the front or side views. Although previous methods have achieved superior performance, they lack the potential for wider applications because they cannot handle complex variations in view distances and view angles in real-world applications. In this study, we specifically focus on addressing the issue of model invariance, aiming to ensure robust performance, despite variations in viewing distance and viewing angles.

Variations in viewing distance can lead to changes in skeletal size. Furthermore, inconsistencies in skeletal size stem from individual differences, such as variations in body type among different individuals and age-related variations in skeletal size. Simple preprocessing techniques cannot adequately address the challenge of action retargeting across different characteristics [9]. Furthermore, the cross-view performance has become an indicator for evaluating models in motion recognition [10, 11]. In motion prediction, the absence of a fixed initial pose for pre-alignment renders cross-view prediction more challenging.

Based on these requirements, the use of three-dimensional (3D) joint rotational angles as the representation of motion is an effective scheme [12, 13]. This scheme represents poses as joints along a kinematic graph and parameterizes the joint orientations as axis angles. However, this ignores the hierarchical structure of the kinematic chain and treats the joints equally [14]. 3D joint coordinates have gradually become more applicable [15, 16] because they address the ambiguities of the rotational angles [17]. The fundamental reason for this ambiguity is that the human perception of movement is based on the spatial position of the joints in 3D coordinates rather than on the rotational angles of the child joints relative to the parent joint. In addition, the mapping from 3D joint coordinates to 3D joint rotation angles is not isometric, leading to differences between the optimal solutions in joint rotation angles and joint coordinates. However, representing motion using joint coordinates introduces irrelevant variables, such as the viewing distance and viewing angle. As shown in Fig. 1, $X_1, X_2$ and $X_3$ share the same motion content. However, owing to variations in the viewing distance and angle, their representations in joint coordinates differ. Consequently, the Euclidean distance between these motion representations is non-zero. Researchers have used complex network designs to achieve view-angle invariance based on 3D joint coordinates [18].

Observation has found that motion represented by 3D joint coordinates contains redundant information, such as skeletal size, which increases the dimensionality of the representation and makes the representation susceptible to changes in view distance. Therefore, we propose using the posture space (PS) to represent motion more compactly and transform the motion into a trajectory on the PS. However, complex manifold optimization techniques are required to achieve trajectory predictions for manifolds. To minimize complexity, we introduce TSRVF [19], which is an isometric transformation between the flattened space and the manifold, implying that we can perform motion trajectory prediction in the flattened space. However, this method cannot achieve view-angle invariance. To overcome these limitations, we propose a new geometric transformation method–specified path transport square-root velocity function (SP-TSRVF) to construct the motion space (MS), which flattens the manifold while eliminating the influences of the view distance and view angle. This approach effectively extracts motion content, provides well-defined distances, and reduces the complexity of optimization. To the best of our knowledge, this is the first study to use Riemannian manifolds to enhance motion prediction invariance. In addition, the metric in MS emphasizes motion evolution, allowing the extraction of common patterns from different motions. Because different motions share these patterns, the complexity of the network can be reduced.

The contributions of this study can be summarized as follows: (1) We proposed a novel framework that combines Riemannian geometry and neural networks for human motion prediction. The introduction of Riemannian geometry provides strong guidance for the learning process, enabling the design of simple networks for motion prediction. (2) We constructed a pre-motion space (pre-MS) to represent motion sequences, effectively separating the skeletal template from the motion content and achieving view distance invariance. The metrics defined by pre-MS focus on the evolution of motions rather than the positions of joints, enabling the effective measurement of distances between motion patterns. (3) We introduce the SP-TSRVF as an isometric mapping between pre-MS and MS. SP-TSRVF achieves view angle invariance by eliminating the view angle transformation group and transforms the nonlinear space problem into a flattened space problem. This transformation allows neural networks to effectively address optimization problems in manifolds without requiring complex manifold optimization techniques.

### Motion representation

There are two widely used representations for motion prediction: 3D joint rotational angles and 3D joint coordinates. Joint rotation representation is used for human motion prediction because 3D joint rotational angles are unaffected by the viewing distance and viewing angle [12, 13, 20]. Some derived representation methods for the rotational angles, such as the quaternion [21] and Stiefel manifold representations [22], have also been proposed. However, Mao et al. [17] discovered flaws in this representation, which has a singularity and cannot distinguish certain motions. Therefore, a 3D joint coordinate representation is used for motion prediction and exhibits superior performance [8, 16]. To avoid stretching artifacts caused by joint representations, Chopin et al. [23] used a cost function to maintain bone consistency. As mentioned earlier, this method is susceptible to changes in the viewing distance and viewing angle. We represented the motion within an MS constructed based on Riemannian geometry. By making the model invariant to variations in view distance and angle, this approach significantly enhances its generalization capabilities.

### Human motion prediction

Various deep-learning methods have been proposed for the resurgence of neural networks. There are four main methods: recurrent neural networks (RNNs), convolutional neural networks (CNNs), graph convolutional networks  (GCNs), and transformers. Fragkiadaki et al. [4] proposed an encoder-recurrent-decoder model in which the recurrent layers incorporate nonlinear encoder and decoder networks, and the motion was predicted in the latent space. Martinez et al. [12] used a sequence-to-sequence architecture to predict the motion sequence. RNN methods have made considerable progress but still suffer from training and discontinuity problems. To address this issue, CNN-based methods [24] were proposed. Liu et al. [7] used 2D convolution to complete the trajectory space transformation, but could not directly model the limb interaction. The GCN is suitable for modeling human motion and numerous GCN-based methods have achieved strong performance in prediction tasks. Ma et al. [8] used a fully connected GCN and achieved higher performance by extracting global spatiotemporal features using temporally and spatially dense GCNs. Cui et al. [25] learned the weights of natural connections and implicit relationships using connective graphs and learnable global graphs, respectively, which increased the flexibility of graph construction. Li et al. [13] designed a multiscale graph to extract features at individual scales and fused them across scales to model the internal relationships of the human body. Dang et al. [26] proposed a novel multiscale residual GCN to extract features from fine to coarse scales, and obtain local and global motion information. However, this method fails to capture the interaction information between the limbs. The transformer can effectively handle sequential data, and this has been validated in natural language processing tasks [27, 28]. Xu et al. [29] combined masking/denoising strategies with a transformer to promote more effective spatiotemporal dependency learning in human motion prediction and achieved excellent performance. However, including auxiliary tasks results in significant resource costs during model training. Some studies have argued that models based on RNNs, CNNs, and GCNs are extremely complex, leading to the emergence of various human motion prediction models based on MLPs [16, 30]. Bouazizi et al. [16] first proposed an MLP-based model called MotionMixer. In this study, a combination of MLPs applied independently to time steps and MLPs applied across body poses were used to extract information and capture the structural and temporal dependencies in motion. Guo et al. [30] discovered that excellent performance can be achieved using an MLP composed of fully connected layers, normalization layers, and transpose operations. However, low-parameter models learn only the mapping between the positions of joints without understanding the meaning of the motion. When the data distribution changed slightly, the performance of the model deteriorated significantly. Our insights suggest that by constructing a novel, reasonable MS to eliminate the variability in data representation, and combining it with an MLP network-based approach, we can achieve effective motion prediction. Therefore, the proposed prediction model has a lower number of parameters (< 0.1 M) and exhibits invariance to both the viewing distance and viewing angle.

### Square-root velocity function

The square-root velocity function (SRVF) was proposed by Srivastava et al. [31] and the elastic metric was calculated by Mio et al. [32]. Bauer et al. [33] summarized the elastic metric as a first-order Sobolev metric and demonstrated its advantages, including reparameterization invariance. This invariance is crucial for modeling human motion because the execution rate of motion can vary. A manifold-aware generative adversarial network (GAN) was proposed to combine the SRVF and GAN to predict motion [23, 34]. These studies drew on shape analysis [31], treating motions as curves in the Euclidean space and transforming them into a hypersphere using SRVF. Subsequently, the motions were mapped to the tangent space of the Karcher mean, and the mapping between the tangent space of historical and future motions was built using a Wasserstein GAN. However, SRVF cannot analyze curves in non-Euclidean spaces because of the inconsistent tangent spaces at each point on the manifold. To address this issue, the transport square-root velocity function (TSRVF) [19], which analyzes curves by transporting the tangent space of the curve along geodesics to a reference point, was proposed. Liu and Zhao [35] successfully applied TSRVF to gesture recognition with favorable results, and Park et al. [36] used it to analyze the action mode. Transporting the tangent space along a geodesic cannot perceive the variance in the viewing angle. Differences in the motion sequence representation caused by changes in viewing angle cannot be eliminated. Thus, we propose the SP-TSRVF, which provides view angle invariance for motion prediction by specifying the path for transporting the tangent space.

The rest of this paper is organized as follows. Method section details the methodology and presents the pseudocode; Results and Discussion section presents the experimental results, including comparisons with other methods, ablation studies, and discussions of the results; Conclusions section concludes the study.

## Methods

In traditional methods, the future pose sequence $X_{N+1:N+T}$ for motion prediction is typically inferred directly from the observed sequence $X_{1:N}=[x_1,x_2,...,x_N]$. In this study, we adopted a different approach by constructing a PS based on the skeletal constraints of the human body. We considered motion as a trajectory on the PS and incorporated shape analysis methods. By removing the view transformation group, we arrive at the final encoding space known as the MS. Leveraging the favorable properties of the encoding space, the proposed method effectively addresses the challenges posed by varying the viewing angles and distances that arise in the practical applications of motion prediction. In addition, using geometric encoding to extract motion information, this method achieves motion prediction using a simple network.

### Overview

**Fig. 2:**
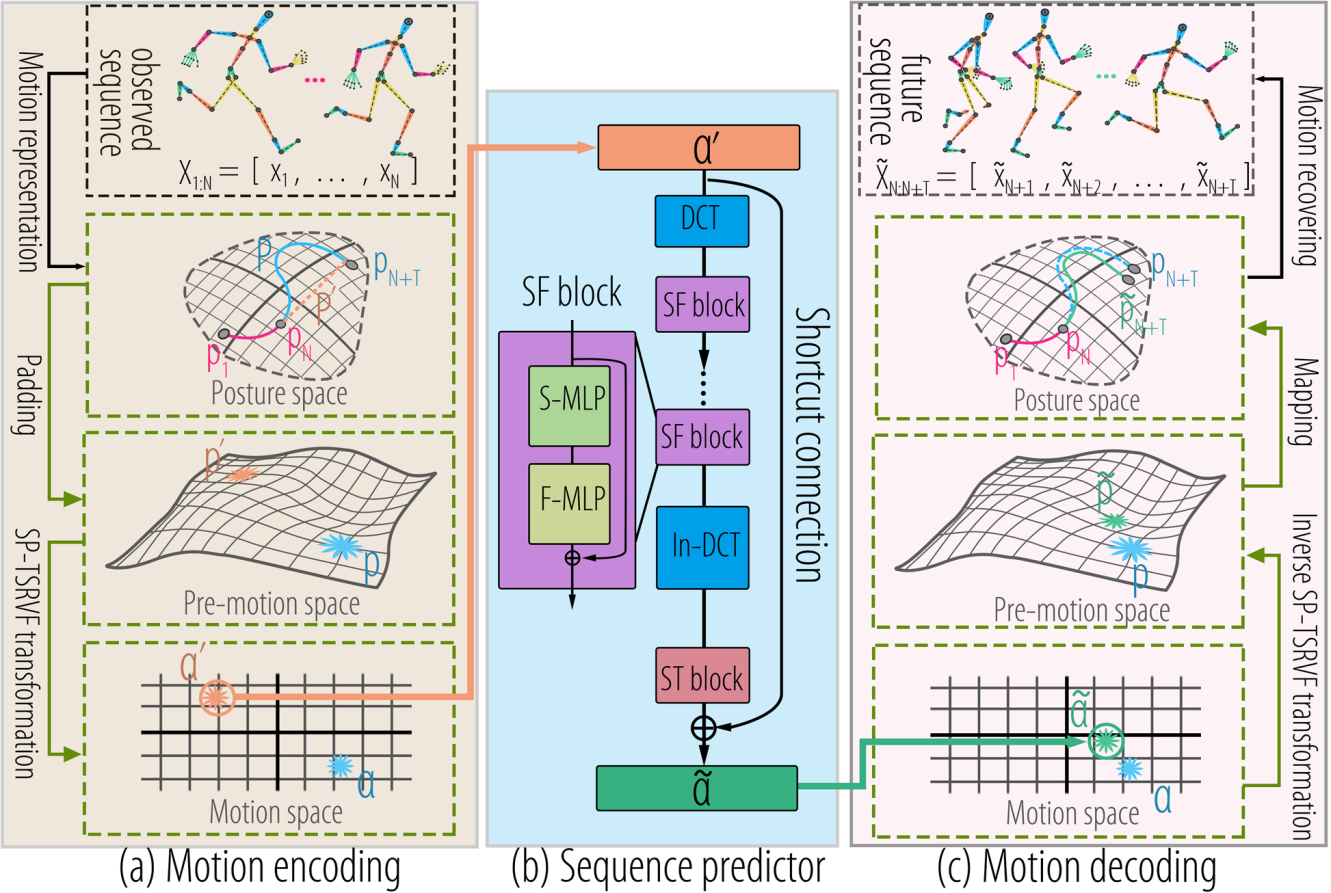
Pipeline of the framework. The network is divided into three components: motion encoding, sequence predictor, and motion decoding. The sequence predictor consists of a shortcut connection, a discrete cosine transform (DCT), spatial-frequency block (SF block), and spatial-temporal block (ST block)

As illustrated in Fig. 2, the proposed framework comprises three main components: encoding, prediction, and decoding. In the encoding phase, the observed sequence is transformed into an MS based on the discrete SP-TSRVF as described in Eq. 7. In the prediction phase, a simple MLP-based network is employed to predict a vector for the input $\alpha ^\prime$, which moves the input to a new position on the MS. In decoding phase, the inverse transformation (Eq. 9), and skeletal templates are used to recover the motion.

**Fig. 3:**
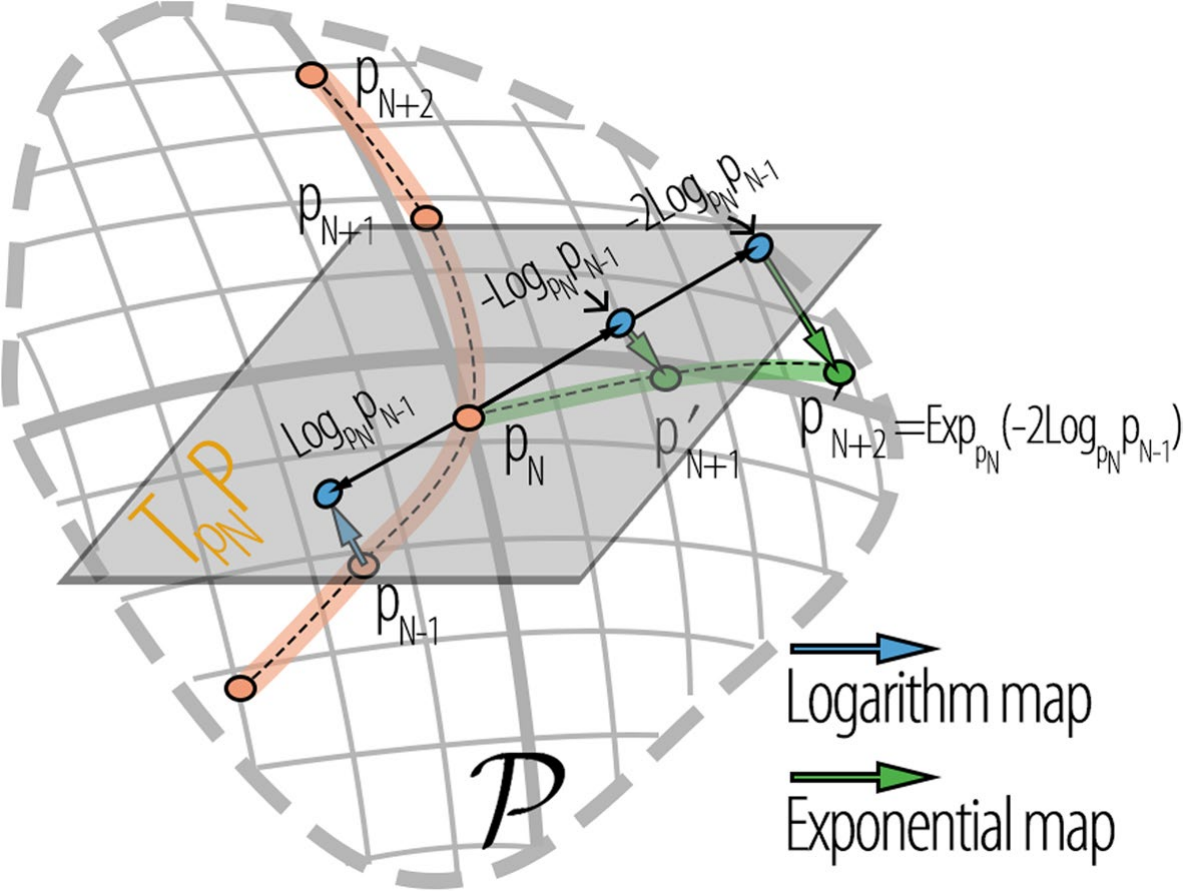
Visualizing padding of motion sequences using motion inertia. $p_N$ is the final frame of the observed sequence, and the motion momentum of $p_N$ is $-\text {Log}_{p_N}{p_{N-1}}$

**Motion encoding.** This component involves padding and encoding the motion sequence $X_{1:N}$ into the MS, as shown in Fig. 2a. Initially, the motion sequence was represented as a trajectory in PS. The observed pose sequence is $P_{1:N}=[p_1,p_2,...,p_{N}]$. As illustrated in Fig. 3, the momentum ${\textbf {Exp}}_{p_N}(-t{\textbf {Log}}_{p_{N}}{p_{N-1}})$ of the motion from the *N*th frame is employed to pad $P_{1:N}$ into a complete sequence $P^\prime _{1:N+T}$. Here, ${\textbf {Exp}}$ and ${\textbf {Log}}$ refer to the exponential and logarithmic maps of PS, respectively, and *t* is the frame index. The padding sequence is as follows:

$$\begin{aligned} P^\prime _{1:N+T} & =\left[p_1^\prime ,p_2^\prime ,...,p_N^\prime ,p_{N+1}^\prime ,...,p_{N+T}^\prime \right]\nonumber \\ & =\left[p_1,p_2,...,p_N,{\text{Exp}}_{p_N}\left(-{\text{Log}}_{p_N}{p_{N-1}}\right),..., {\text{Exp}}_{p_N}\left(-T{\text{Log}}_{p_N}{p_{N-1}}\right)\right]\end{aligned}$$

The padding sequence $P^\prime _{1:N+T}$ and ground truth $P_{1:N+T}$ are represented as ${p}^\prime$ and *p*, respectively, in pre-MS. Finally, the SP-TSRVF encodes ${p}^\prime$ and *p* into the MS, which is a flattened space with $\mathbb {L}^2$. The encoded results are represented as $\alpha ^\prime$ and $\alpha$.

**Sequence predictor.** This component predicts the sequence $\tilde{\alpha }$ based on input $\alpha ^\prime$. It uses a simple network architecture consisting of an MLP, as illustrated in Fig. 2b. To capture shared information regarding motion changes, we incorporated shortcut connections into the network. These shortcut connections enable the network to learn how to shift the input sequence $\alpha ^\prime$ toward ground truth $\alpha$. The presence of shortcut connections also contributes to the stability of network training. By adopting a more compact representation of the motion, the dimensionality of the solution space is compressed, that is, from $\mathbb {R}^{T\times (K+1)\times 3}$ to $\mathbb {R}^{(T-1)\times K\times 2}$, where $K+1$ is the number of joints. In addition, using an evolutionary metric as a loss function forces the network to learn repetitive motion patterns. Therefore, this MLP-based network for motion prediction has fewer than 0.1 M parameters.

**Motion decoding.** As shown in Fig. 2c, this component serves as the inverse of the encoding process. It employs the inverse SP-TSRVF to restore the predicted sequence $\tilde{\alpha }$ to ${\tilde{p}}$. Next, ${\tilde{p}}$ is mapped back to the PS, yielding sequences $[\tilde{p}_{N+1},\tilde{p}_{N+2},...,\tilde{p}_{N+T}]$. Finally, using the skeletal template from the *N*-th frame, the motion sequence is recovered back to the 3D joint coordinates, denoted $\tilde{X}_{N+1:N+T}$.

### View distance invariant representation on pre-MS

**Fig. 4:**
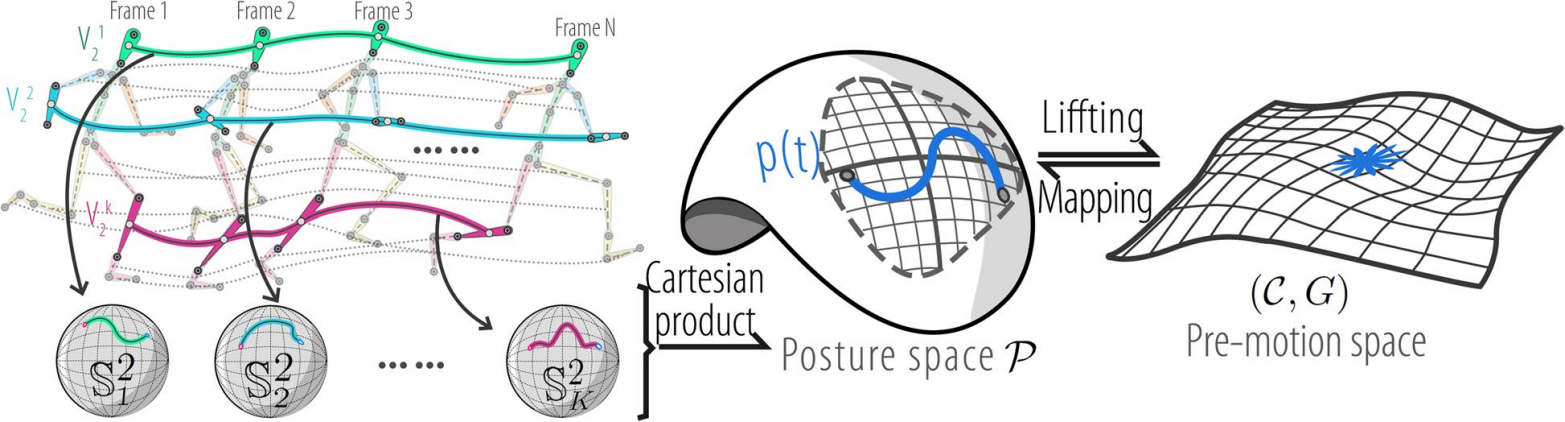
Illustration of space construction. PS is the product manifold of K $\mathbb {S}^2$, denoted as $\mathcal {P}$. The pre-MS is denoted as $(\mathcal {C},G)$

To achieve a view distance-invariant representation of the motion, we separate the skeletal template from the motions and construct the pre-MS. This is illustrated in Fig. 4.

**PS.** The position of the *i*th joint in 3D coordinates at time *t* is denoted as $x_{i}(t)$. The unit directional vector connecting the *i*th joint to the *j*th joint of the bone is represented as follows:

1 $$\begin{aligned} \text{s}_{k}(t)=\frac{x_{i}(t)-x_{j}(t)}{l_k(t)}\in \mathbb {S}^2, l_k(t)=||x_{i}(t)-x_{j}(t)||_{2} \end{aligned}$$

where $\textbf{s}_{k}$ is the unit directional vector of the *k*th bone, $l_k$ is the length of *k*th bone at time *t*. As the length of the skeleton does not change over time, the motion of the *i*th joint $x_{i}(t)\in \mathbb {R}^3$ can be represented by $\mathbb {S}^2$ (unit sphere).

#### Definition 1

(Posture space). The PS $\mathcal {P}$ is a product manifold that is composed of the $\mathbb {S}^2$ manifold through Cartesian product. The posture of the skeleton with *K* bones is denoted by $\mathcal {P}:\mathbb {S}^2_1\times \mathbb {S}^2_2\times ...\times \mathbb {S}^2_{K}$.

**Fig. 5:**
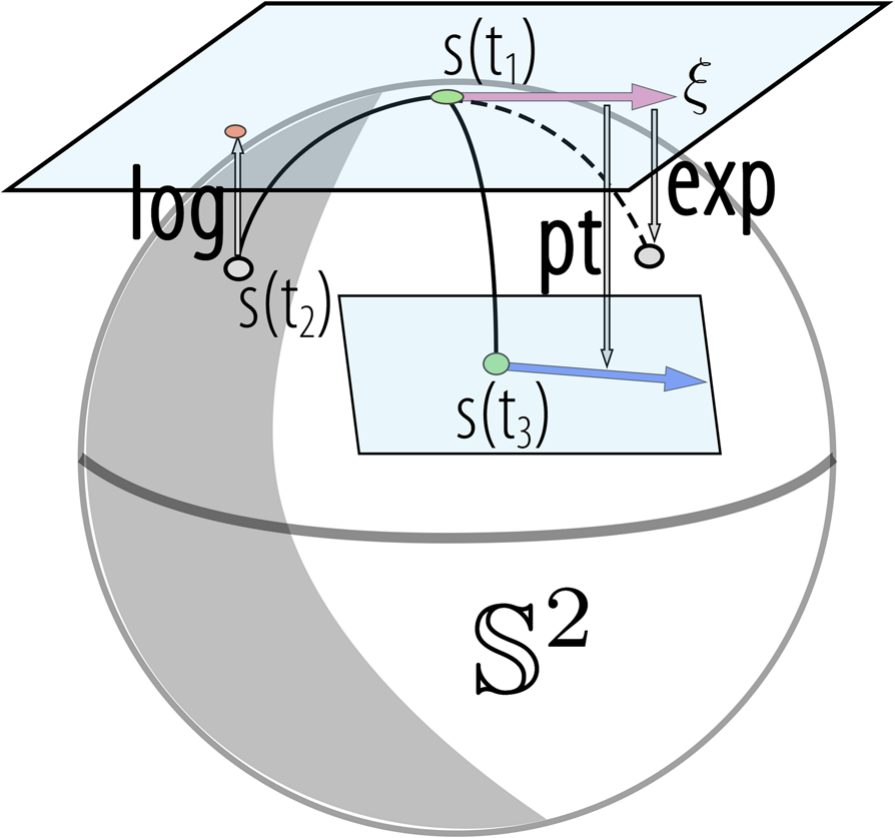
Illustration of the exponential map, logarithmic map, and parallel transport on $\mathbb {S}^2$ manifold

The basic geometric tools for the PS were constructed using the tools of the ingredient space $\mathbb {S}^2$. As shown in Fig. 5, the exponential map of $\mathbb {S}^2$ is $\exp _{s(t_1)}(\xi )=\cos (||\xi ||_2)s(t_1)+\frac{\sin (||\xi ||_2)}{||\xi ||_2}\xi ,$ the logarithmic map of $\mathbb {S}^2$ is $\log _{s(t_1)}(s(t_2))=\frac{\rho }{\sin (\rho )}(s(t_2)-s(t_1)\cos (\rho )),$ and the parallel transport from $s_{i}$ to $s_{j}$ on $\mathbb {S}^2$ is denoted as $pt_{s(t_1)\rightarrow s(t_2)}(\xi )=\xi -\frac{\langle s(t_2),\xi \rangle }{1+\cos (\rho )}(s(t_1)+s(t_2)),$ where $s(t_1),s(t_2), s(t_3)\in \mathbb {S}^2$, $\xi \in T_{s(t_1)}\mathbb {S}^2$; $\rho$ denotes the Riemannian distance in $\mathbb {S}^2$; and $\langle \cdot ,\cdot \rangle$ denotes the Euclidean inner product.

The exponential map, logarithm map, and parallel transport of PS are denoted as ${\textbf {Exp}}_{p(t_1)}(\Xi )$, ${\textbf {Log}}_{p(t_1)}(p(t_2))$, and ${\textbf {Pt}}_{p(t_1)\rightarrow p(t_2)}(\Xi )$ respectively.

2 $$\begin{aligned} {\text{Exp}}_{p(t_1)}(\Xi ) & =(\exp _{s_{1}(t_1)}(\xi _{1}),\exp _{s_{2}(t_1)}(\xi _{2}),...,\exp _{s_{K}(t_1)}(\xi _{K})) \end{aligned}$$

3 $$\begin{aligned} {\text{Log}}_{p(t_1)}(p(t_2)) & =(\log _{s_{1}(t_1)}(s_{1}(t_2)),\log _{s_{2}(t_1)}(s_{2}(t_2)),...,\log _{s_{K}(t_1)}(s_{K}(t_2))) \end{aligned}$$

4 $$\begin{aligned} {\text{Pt}}_{p(t_1)\rightarrow p(t_2)}(\Xi ) & =(pt_{s_{1}(t_1)\rightarrow s_{1}(t_2)}(\xi _{1}),...,pt_{s_{K}(t_1)\rightarrow s_{K}(t_2)}(\xi _{K})) \end{aligned}$$

where $p(t_1),p(t_2)\in \mathcal {P}$, $\Xi \in T_{p(t_1)}\mathcal {P}$, $T_{p(t_1)}\mathcal {P}$ is the tangent space of $p(t_1)$. Currently, each pose of motion is represented in PS, and this representation remains unaffected by the viewing distance. We considered these motions as trajectories within the PS and performed a geometric analysis of these trajectories.

#### Definition 2

(Trajectories set). The set of motion trajectories on $\mathcal {P}$ as $\mathcal {C}=\{{p}\in AC(I,\mathcal {P})|{p}^\prime (t)\ne 0, \forall t\in I \}$, where $AC(I,\mathcal {P})$ refers to the collection of absolutely continuous curves that have a domain in *I* and a range in $\mathcal {P}$, and *t* is time parameter.

We propose that the distance of motion should be defined by variations in motion evolution, specifically by considering changes in direction and speed rather than the absolute position of joints in space. Using the motion evolution distance, the model can increase its robustness to the initial posture and improve its ability to learn motion patterns. Therefore, we suggest the following equation for pre-MS:

5 $$\begin{aligned} G_{{p}}(\xi , \zeta )=\int _I g\underbrace{\left( \nabla _{s} \xi ^\perp , \nabla _{s} \zeta ^\perp \right) }_{\text {speed difference}}+ \frac{1}{4}g\underbrace{\left( \nabla _{s} \xi ,\partial _s{p} \right) g\left( \nabla _{s} \zeta ,\partial _s{p} \right) }_{\text {direction difference}}\textrm{d}s \end{aligned}$$

where $\xi ,\zeta \in T_{{p}}\mathcal {P}$, $\partial _s {p}$ is the unit length tangent vector along *p*, $\textrm{d}s=\Vert {p}^\prime (t)\Vert _2\textrm{d}t$ denotes arc length integration, and is the vertical component of $\nabla _{s} \xi ^\perp =\nabla _{s} \xi -g(\nabla _{s} \xi ,\partial _s{p})\partial _s{p}$. Equation 5 is a first-order Sobolev equation. In this equation, $g\left( \nabla _{s} \zeta ,\partial _s{p} \right)$ represents a projection along the tangential direction of *p*, which quantifies the change in speed resulting from variations in motion. $\nabla _{s} \xi ^\perp$ captures the changes orthogonal to the speed by measuring the variations in the direction of motion caused by the motion changes.

#### Definition 3

(Pre-motion). The pre-MS is a Hilbert space that is the trajectories set $\mathcal {C}$ equipped with the metric *G*. The pre-MS is denoted as $(\mathcal {C}, G)$.

**Fig. 6:**
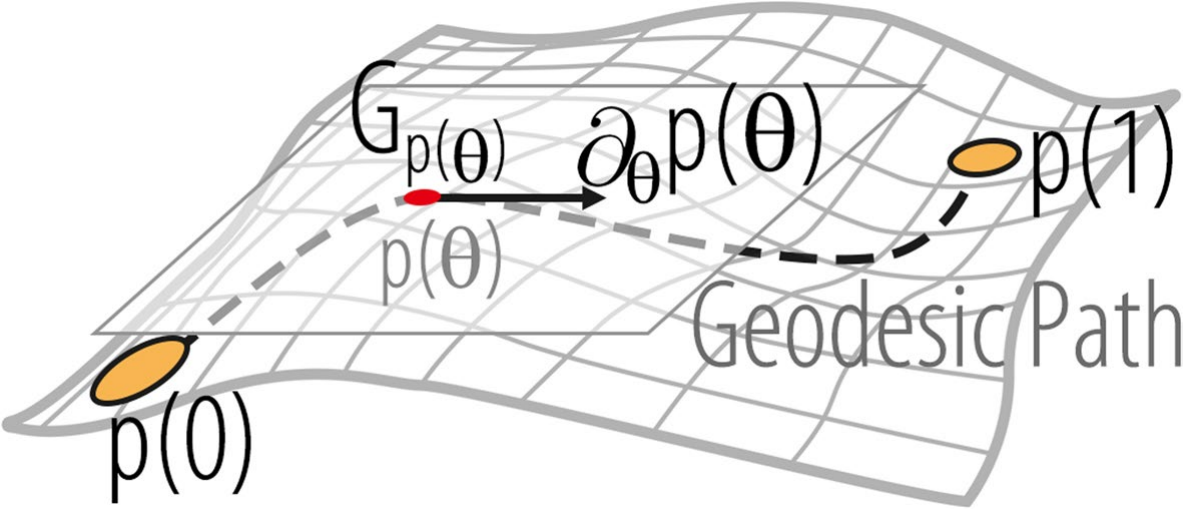
Visualization of the geodesic path on the pre-MS

The geodesic distance in pre-MS is defined as the infimum over the lengths of all paths:

6 $$\begin{aligned} dist_\mathcal {C}({p}(0),{p}(1))=\inf _{{p}(\theta )}\int _0^1\sqrt{G_{{p}(\theta )}(\partial _\theta {p}(\theta ),\partial _\theta {p}(\theta ))}d\theta \end{aligned}$$

where ${p}(\theta )$ is short for ${p}(\cdot ,\theta )$, which represents the path-connecting motions *p*(0) and *p*(1). Note that $dist_{\mathcal {C}}(\cdot ,\cdot )$ is view-distance invariance. The length of the geodesic path is referred to as the geodesic distance. The geodesic path in pre-MS space is shown in Fig. 6.

Computing the geodesic distance on the manifold is an extremely complex Riemannian optimization problem, as shown in Eq. 6. The considerable computational and time costs involved in computing the distance between the output and ground truth severely impact the training of the sequence predictor. According to ref. [19], TSRVF is an isometric mapping from ($\mathcal {C},G_{{p}}$) to the flattened space. Therefore, it is possible to transform the pre-MS into a flattened space using TSRVF mapping. The TSRVF is

$$\Psi _{TSRVF}({p})=\alpha (t)=\frac{{p}^\prime (t)_{{p}(t)\rightarrow {p}(0)}}{\sqrt{||{p}^\prime (t)||_2}}\in T_{{p}(0)}\mathcal {P}$$

**Fig. 7:**
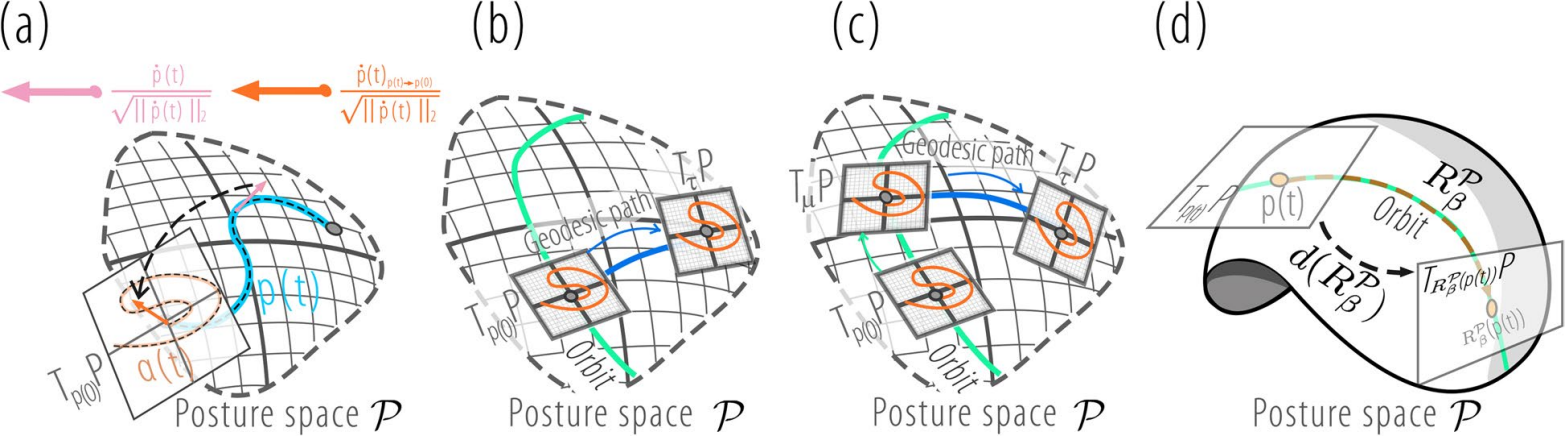
Illustration of TSRVF, SP-TSRVF, and group action. **a** Generation and parallel transport of tangent vectors; **b** TSRVF aligning the tangent space along the geodesics; **c** SP-TSRVF aligning the tangent space along a specified path; **d** The group $R^{\mathcal {P}}_{\beta }$ action on PS

$\Psi _{TSRVF}$ is shown in Fig. 7a. $\alpha (t) \in T_{p(0)}\mathcal {P}$ is parallel to the geodesic from *p*(0) to the tangent space $T_\tau \mathcal {P}$, where $\tau$ is the unified reference point for all the motions, as shown in Fig. 7b.

### View angle invariant representation on MS

**Fig. 8:**
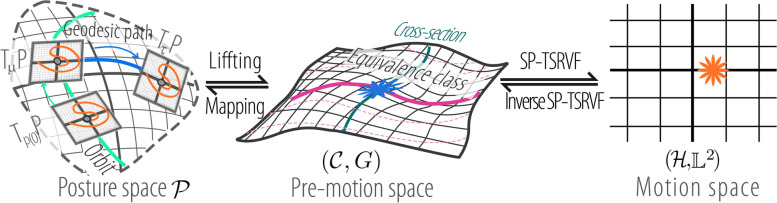
Illustration of MS construction. $\tau$ is the reference point in PS $\mathcal {P}$, $T_\tau \mathcal {P}$ is the tangent space of $\tau$. The MS is denoted as $(\mathcal {H},\mathbb {L}^2)$. The green trajectory in PS is the orbit of $\widetilde{SO}(\mathcal {P})$. The pink path in pre-MS represents an equivalence class of a motion, and it will be mapped to the orange point on MS using SP-TSRVF

In real-world scenarios, as shown in Fig. 1, the view angle may vary. This leads to the existence of an equivalence relation, such as view angle variations, on the pre-MS, as shown in Fig. 8. That is, the pre-MS was constructed using the equivalence class of motion. However, by aligning the tangent spaces of motions directly along geodesic paths, as in TSRVF, cannot eliminate this equivalence relationship.

The view angle changes by an angle $\beta$, causing the positions of the joints represented in the 3D joint coordinates to rotate around the z-axis by the same angle $\beta$, that is, $R_{\beta }(x(t))=[R(\beta )x_{1}(t),R(\beta )x_{2}(t),...,R(\beta )x_{K+1}(t)].$ Consequently, the view-angle transformation group on PS is

$$\begin{aligned} \widetilde{SO}(\mathcal {P})= \left[ \begin{array}{cccc} \widetilde{SO}(2) & & & \\ & \widetilde{SO}(2)& & \\ & & ...& \\ & & & \widetilde{SO}(2) \end{array} \right] \subset SO(3\times K),\widetilde{SO}(2)\subset SO(3) \end{aligned}$$

The action of the view-angle transformation group on element $p_t$ in the PS can be denoted as follows: $R^{\mathcal {P}}_{\beta }({p}(t))=[R(\beta ){s}_{1}(t),R(\beta ){s}_{2}(t),...,R(\beta ){s}_{K}(t)]$. As shown in Fig. 7d, the group of view angle transformations can transform the point *p*(*t*) to another point $R^{\mathcal {P}}_{\beta }({p}(t))$ within the orbit. The differential of the group action can transform the tangent space $T_{p(t)}\mathcal {P}$ into another tangent space $T_{R^{\mathcal {P}}_{\beta }({p}(t))}\mathcal {P}$ within the orbit.

#### Proposition 1

Let $\widetilde{SO}(\mathcal {P})$ be a group action on set $\mathcal {P}$. If the group element $R^{\mathcal {P}}_{\beta }\in \widetilde{SO}(\mathcal {P})$ transforms the point $p_t$ to the point $R^{\mathcal {P}}_{\beta }(p(t))$, then the differential of the group action $d(R^{\mathcal {P}}_{\beta })$ transforms the tangent space from the point $p_t$ to the point $R^{\mathcal {P}}_{\beta }(p(t))$, i.e.,

$$\begin{aligned} d\left(R^{\mathcal {P}}_{\beta }\right):v\in T_{p(t)}\mathcal {P}\mapsto R^{\mathcal {P}}_{\beta }(v)\in T_{R^{\mathcal {P}}_{\beta }(p(t))}\mathcal {P}, R^{\mathcal {P}}_{\beta } \in \widetilde{SO}(\mathcal {P}) \end{aligned}$$

#### Definition 4

(Specified path transport square-root velocity function). The SP-TSRVF is

7 $$\begin{aligned} \Psi _{SP-TSRVF}({p})(t)=\alpha (t)=\frac{d\left(R^{\mathcal {P}}_{{p}(0)\rightarrow \mu }\right)\left({p}^\prime (t)_{{p}(t)\rightarrow {p}(0)}\right)_{\mu \rightarrow \tau }}{\sqrt{||{p}^\prime (t)||_2}}\in T_{\tau }\mathcal {P}\end{aligned}$$

where $d\left(R^{\mathcal {P}}_{{p}(0)\rightarrow \mu }\right)$ represents the alignment of the tangent space at point *p*(0) with that at point $\mu$.

When encoding using $\Psi _{SP-TSRVF}$, the value of $\mu$ is determined by finding the point on the orbit of *p*(0) that is closest to the reference point $\tau$: $\mu =\min _{\mu ^*\in \widetilde{SO}(\mathcal {P})\cdot p(0)} d_R(\tau , \mu ^*).$ The choice of reference point $\tau$ is arbitrary. For convenience, the Riemannian mean of the initial poses on the PS in the training set was selected as the reference point. Since all the tangent spaces have been moved along the orbit to their respective $\mu _m$, it is equivalent to having all the motions lying on the cross-section of the pre-MS, as shown in the middle of Fig. 8. In this case, the representation of the motion is independent of the viewing angle. Consequently, $\Psi _{SP-TSRVF}$ encodes motions with shared content but different viewing angles at the same location on the MS.

#### Definition 5

(Motion space). Let the $\Psi _{SP-TSRVF}(\cdot )$ be the SP-TSRVF transformation. The set of curves in $T_\tau \mathcal {P}$ is denoted $\mathcal {H}=\left\{ \alpha \in L^2(I,T_\tau \mathcal {P}) |\alpha =\Psi _{SP-TSRVF}({p}) \right\}$, where $L^2(I,T_\tau \mathcal {P})$ is the set of $L^2$ integrable functions. The $\mathbb {L}^2$-metric is equipped with $\mathcal {H}$. The Hilbert space $(\mathcal {H}, \mathbb {L}^2)$ is referred to as the MS.

According to the Definition (5), the distance between motions $\alpha _1$ and $\alpha _2$ is calculated by

8 $$\begin{aligned} dist_\mathcal {H}({[X_1],[X_2]})=dist_\mathcal {H}({\alpha _1,\alpha _2}) =\sqrt{\int _0^1 \Vert {\alpha _1(t)-\alpha _2(t)} \Vert ^2_2 dt}\end{aligned}$$

Based on the distances in Eq. 8, the following property holds: As shown in Fig. 1, $X_1$ and $X_2$ represent two motions belonging to the same equivalence class [*X*]. Although they share identical content, they differ in terms of viewing distance and angle. Specifically, $X_1 = R^{\mathcal {P}}_{\beta }(aX_2)$, where $a\in \mathbb {R}^+, R_\beta \in \widetilde{SO}(\mathcal {P})$. When considering the mean per joint position distance, a non-zero distance exists between $X_1$ and $X_2$. However, after encoding these motions into the MS, the distance between them becomes zero. This property demonstrates that MS achieves invariance in both viewing distance and viewing angle.

Overall, to encode motion into MS, the PS, and pre-MS are introduced as auxiliary spaces for encoding. We first transform each frame of motion into a PS through a skeleton normalization method, which eliminates the influence of viewing distance and individual skeletal differences because the skeletons are normalized. To enable the analysis of curves on manifolds, we considered the manifold curve as an element in the pre-MS. However, pre-MS contains equivalent classes of view angle variations, meaning that the motion expression in pre-MS is affected by view angle variations. We found that the pre-MS is a homogeneous space of the view angle transformation group, and by using parallel transport to align the tangent spaces of different motions along a specific path, the expression differences caused by view angle variations are eliminated. Therefore, SP-TSRVF is proposed, which moves along a specified path (‘**orbit** “+” **geodesic**’) rather than a geodesic path when aligning the tangent spaces with the reference point, ultimately forming a space (MS) that does not contain equivalence classes of view-angle variations. The motion representation on the MS is invariant to variations in the viewing distance and viewing angle.

### Inverse SP-TSRVF

When $\alpha (t)$ is transported from $\tau$ along the path opposite to the specified path to *p*(0), it becomes a vector-valued curve in $T_{{p}(0)}\mathcal {P}$. An integral curve for $\alpha (t)$ with the initial condition *p*(0) is a trajectory of $\mathcal {P}$, $\tilde{{p}}: I\rightarrow \mathcal {P}$ such that

9 $$\begin{aligned} \frac{d\tilde{{p}}(t)}{dt}=\alpha (t)\Vert \alpha (t)\Vert _{2,{p}(0)\rightarrow \tilde{{p}}(t)} \end{aligned}$$

The space commutative diagram between pre-MS and MS is shown in Fig. 8.

### Implementation of SP-TSRVF and inverse SP-TSRVF

In motion-prediction tasks, it is necessary to perform geometric encoding on discrete sequences $X_{1:N}=[x_1,x_2,...,x_N]$. According to Eq. 1, the historical sequence $X_{1:N}$ is transformed into a PS denoted by $P_{1:N}=[p_1,p_2,...,p_N]$. The bone lengths at time N were recorded as the skeleton template and represented as $L_N=[l_{1,N},l_{2,N},...,l_{K,N}]$ at time *N*. To represent the differential operator for a motion sequence in discrete form, we denote this as ${\textbf {Diff}}(\cdot )$. The discrete forms of Eq. 7, based on Eqs. 4 and 3 is

$$\begin{aligned} \Psi _{SP-TSRVF}(p_i) & ={\text{Pt}}_{\mu \rightarrow \tau }\left(R^\mathcal {P}_\beta \left({\text {Pt}}_{p_2\rightarrow p_1}\left(...\left({\text{Pt}}_{p_i\rightarrow p_{i-1}}\left( \frac{{\text{Diff}}(p_i)}{\sqrt{||{\text{Diff}}(p_i)||_2}}\right)\right)\right)\right)\right)\\ & ={\text{Pt}}_{\mu \rightarrow \tau }\left(R^\mathcal {P}_\beta \left({\text{Pt}}_{p_2\rightarrow p_1}\left(...\left({\text{Pt}}_{p_i\rightarrow p_{i-1}}\left( \frac{{\text{Log}}_{p_i}(p_{i+1})}{\sqrt{||{\text{Log}}_{p_i}(p_{i+1})||_2}}\right)\right)\right)\right)\right) \end{aligned}$$

The unified encoded ${P}^\prime _{1:N+T}$ is represented as $\alpha ^\prime =[\alpha ^\prime _1,\alpha ^\prime _2,...,\alpha ^\prime _{N+T}]$, as shown at the bottom of Fig. 2a. The ground truth $P_{1:N+T}$ is mapped to the MS using the same method and is denoted by $\alpha =[\alpha _1,\alpha _2,...,\alpha _{N+T-1}]$. The discrete form of the inverse SP-TSRVF is

$$\begin{aligned} \Psi ^{-1}_{SP-TSRVF}({\alpha }_i)={\text{Exp}}_{\text{p}_{i}}\left({\text{Pt}}_{p_{i-1}\rightarrow p_{i}}\left(...\left({\text{Pt}}_{p_{1}\rightarrow p_{2}}\left(R^\mathcal {P}_{-\beta }\left({\text{Pt}}_{\tau \rightarrow \mu }\left({\alpha }_i||{\alpha }_i||_2\right)\right)\right)\right)\right)\right) \end{aligned}$$

The output of the predictor is denoted by $\tilde{\alpha }$, and it must be mapped onto a trajectory on the PS using the inverse SP-TSRVF. The resulting trajectory is denoted as $\tilde{{P}}_{1:N+T}$. As illustrated at the top of Fig. 2c, the prediction $\tilde{P}_{N+1:N+T}$ and skeleton template $L_N$ are used to recover the motion sequence into 3D joint coordinates $\tilde{X}_{N+1:N+T}$. This is achieved by updating each joint position $x_{i,child}=x_{i,parent}+p_{i,k}\times l_{N,k}$, where $x_{i,parent}$ and $x_{i,child}$ represent the connected joints of bone *k* in a parent-child order, $N+1\le i\le N+T,i\in \mathbb {Z}$. Details of the SP-TSRVF and inverse SP-TSRVF transformation are presented in Algorithms 1 and 2.

**Complexity analysis.** The proposed method achieves view distance and view angle independent motion prediction by constructing a manifold that can compactly represent the motion. However, the SP-TSRVF transforms the manifold into a flattened space, thus avoiding the complexity of manifold optimization. Next, the computational complexity of the SP-TSRVF transformation algorithm was analyzed. The constructed manifold is based on a hypersphere, and its operators, such as exponential map, logarithmic map, and parallel transport, have analytical solutions. Therefore, the ${\textbf {Diff}}(\cdot )$, ${\textbf {Pt}}(\cdot )$ and ${\textbf {Exp}}(\cdot )$ operations in Algorithms 1 and 2 can be computed analytically with complexity *O*(1). The distance function $d_R(\tau , R^\mathcal {P}_{\beta }(c(0))$ is convex and has only one variable; thus, $\beta$ can be solved analytically with a complexity of *O*(1). The main computational cost of the algorithm originates from the nested loops transported in parallel along the specified path. The complexity of this loop is related to the number of observed frames *N* and the number of future frames $T-1$, that is $O((N+T)*(N+T)/2)$. Therefore, the overall complexity of the encoding and decoding components of the algorithm is approximately $O((N+T)^2)$.

**Figure:**
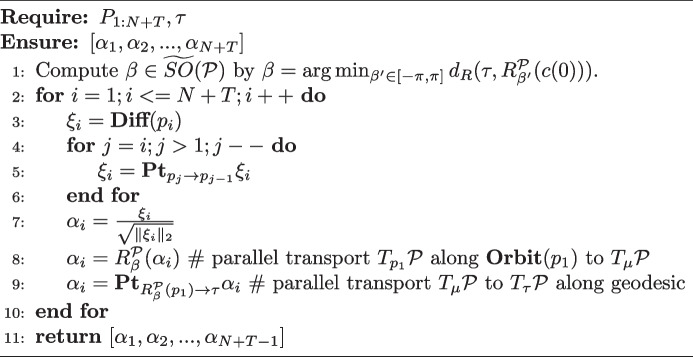
**Algorithm 1** SP-TSRVF transformation

**Figure:**
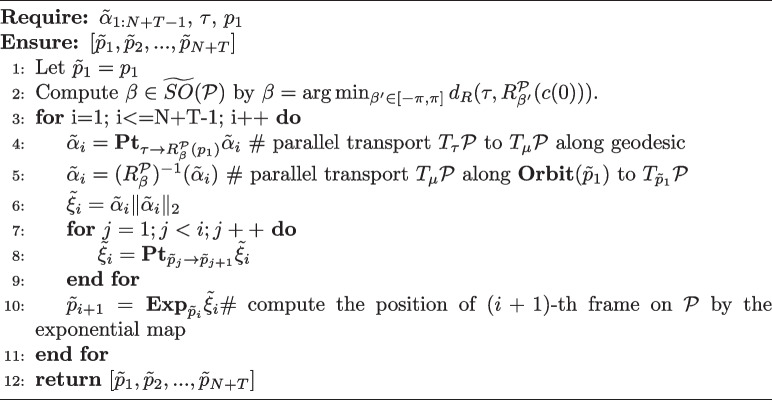
**Algorithm 2** Inverse SP-TSRVF transformation

### Network architecture

**Fig. 9:**
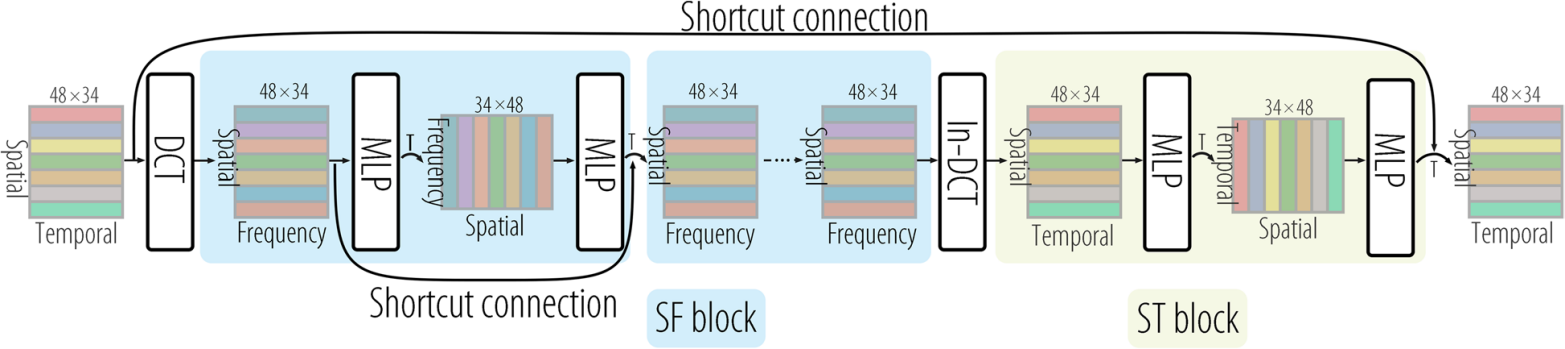
Network structure diagram

In Fig. 2b, the network is based on the residual concept of ResNet [37, 38]. Figure 9 provides details regarding the structure of the network. Assuming a motion prediction task for a skeleton with 25 joints, the input data $X\in \mathbb {R}^{75\times 10}$ are encoded as $\alpha ^\prime \in \mathbb {R}^{48\times 34}$ after padding and SP-TSRVF encoding. Padding implies the initial assumption that motion will continue in the direction and speed of the last frame. Therefore, the part within the shortcut connections learns to move the initial assumption $\alpha ^\prime$ on the MS toward the ground truth $\alpha$. The network learns the displacement vectors rather than the positions on the MS. This suggests that the network has acquired reusable features, which aligns with the repetitive motion patterns often seen in movements like standing, sitting, and arm waving. Therefore, the use of shortcut connections can stabilize future motions. To capture the temporal correlation of the motions, DCT was employed to extract the motion components. The network includes an SF block that consists of a spatial MLP (S-MLP) and a frequency MLP (F-MLP). S-MLP, with one hidden layer, establishes relationships between body joints, whereas F-MLP, with one hidden layer, establishes relationships between motion components. The ST block captures relationships in both the spatial and temporal domains using two linear layers. Utilizing the distance function (Eq. 8), the network loss is defined as follows:

$$\begin{aligned} \mathcal {L}=\sqrt{\sum \limits _{k=1}^K\sum \limits _{t=N}^{N+T-1}\frac{1}{(t-N+1)^\gamma }\Vert \tilde{\alpha }_{t,k}-\alpha _{t,k}\Vert ^2_2} \end{aligned}$$

where $\gamma$ is used to balance the cumulative error of time.

## Results and Discussion

To verify the performance of the proposed method and its invariance to viewing distance, we tested it on Human 3.6M and CUM MoCap. The human 3.6M dataset is characterized by an abundant amount of data and high-quality motion. The characteristic of the CUM MoCap dataset is that it has fewer samples but more violent motions. All experiments are performed on an off-the-shelf computer with an Intel(R) Core(TM) i9-9900K 3.60GHz processor, 64 GB of RAM, and a GeForce RTX 3090 graphics card. This study uses the *Geomstats* [39] library.

### Datasets and evaluation

**Human 3.6M.** Following previous research, we used the Human 3.6M [40] dataset, which has 15 types of action performed by seven actors (S1, S5, S6, S7, S8, S9, and S11). The first protocol considered 17 joints after excluding joints with constant readings or close to others following refs. [23, 34]. In the second protocol, 22 joints were included for each pose as an exponential map. These were converted into 3D coordinates following previous studies [16, 17], omitting ten redundant joints. The frame rate was downsampled from 50 to 25 fps, and global rotations and translations of poses were excluded following previous studies. S5 was used for testing. The skeletal templates of the synthetic test data were scaled using a Gaussian distribution $\mathcal {N}(1,0.5)$ and randomly rotated around the z-axis within the range $[-\pi ,\pi ]$ to demonstrate the proposed method’s view distance and view angle invariance.

**CMU MoCap.** The CMU MoCap dataset contains 3D skeletal motion data with 40 objects under multiple infrared cameras. 3D coordinate representations and a training/test split, as in Mao et al. [17] were adopted. Based on previous studies [16, 23, 30], eight actions were used for training and testing, and 25 and 17 joints were reserved. The other preprocessing strategies matched those used for the Human 3.6M dataset.

**Evaluation metrics.** The standard mean per joint position error (MPJPE) (Eq. 10) was used to measure the performance of the following approaches [40]: MPJPE compares the 3D coordinates of the predicted sequence with those of the ground-truth sequence in specified milliseconds.

10 $$\begin{aligned} \text {MPJPE}=\frac{1}{K}\sum \limits _{k=1}^K\Vert \tilde{x}_{t,k}-x_{t,k}\Vert _2 \end{aligned}$$

AMPJPE (Eq. 11) is the average MPJPE for 0-1000 ms. AMPJPE is used to measure the overall performance of the model.

11 $$\begin{aligned} \text {AMPJPE}=\frac{1}{T}\frac{1}{K}\sum \limits _{t=N+1}^{N+T}\sum \limits _{k=1}^K\Vert \tilde{x}_{t,k}-x_{t,k}\Vert _2 \end{aligned}$$

**Implementation details.** The input length was 10, and the output was 25 for Human 3.6M and CMU MoCap datasets. We followed the study by Dang et al. [26] for the entire test dataset and used the Adam optimizer as a solver for training the model. The initial learning rate was 0.005 for the Human 3.6M dataset and 0.001 for the CMU MoCap dataset. The decay weight was configured as 1e-4 for both datasets. The model was trained to achieve optimal performance for 200 epochs on the Human 3.6M dataset and 600 epochs on the CMU MoCap dataset. A batch size of 512 was employed during the training.

**Comparison experiment.** In the first protocol, we compare the proposed method with three baselines, zero-velocity [12], LDRGCN [25], and manifold-aware GAN [23]. Because the experiments in this protocol are based on ref. [23], the results are cited from that paper. In the second protocol, we compared the proposed method with eight baseline methods on both standard and synthetic datasets. The baselines included the DMGNN [13] and MMixer-Euler [16], which are representative models that utilize rotation angles. We also evaluated the performance of LTD [17] and PGBIG [8], which are representative models known for their high accuracy in motion prediction. In addition, we considered the STS-GCN [15], MMixer-3D [16], and siMLPe [30], which are representative models for simple networks. Zero-velocity [12] was used as a baseline to evaluate whether the model functioned properly.

### Results of Human 3.6M

We validate the effectiveness of the proposed method through experiments on a standard dataset and verify its invariance on a synthetic dataset. Ablation experiments were conducted to validate the view distance and view angle invariance of geometric encoding, while also assessing the contributions of various network blocks to the performance. To evaluate the performance of the model, we conducted tests on standard datasets (Tables  1, 2, and 3) and synthetic test datasets (Table 4).

In the first protocol, the proposed method achieved state-of-the-art performance for both short- and long-term predictions (Table 1). We compared our method with a manifold-aware GAN, which also uses SRVF geometric encoding, but introduces distortion through logarithmic mapping over long distances. The proposed method avoids this distortion by transporting the tangential vectors along the trajectory in parallel. In addition, the proposed method preserves the amplitude information of the movements, unlike the manifold-aware GAN, which scales the length of the motion trajectories to the unit hypersphere. Consequently, the proposed method outperformed the manifold-aware GAN with an 8.2$\%$ reduction in MPJPE at 1000 ms. To demonstrate the performance of the proposed method, we compared it with the rotation-angle-based approach (DMGNN, MMixer-Euler). The results predicted by the comparative method were reconstructed into 3D joint coordinate representations. In Table 2, we observe that the proposed method achieves a reduction of $17.5\%,8.5\%,5.5\%$, and $4.4\%$ at 80, 160, 320, and 400 ms in MPJPE, respectively. These results highlight the inadequacy of rotational angles in accurately quantifying joint position errors in 3D joint coordinates.

In the second protocol (Table 3), the proposed method demonstrated good continuity and competitive performance at 80 and 160 ms, particularly compared with the low-parameter (parameter< 0.2 M) models. PGBIG, which is based on GCN, achieves state-of-the-art performance in both short- (less than 400 ms) and long-term predictions (400-1000 ms) by leveraging the information of the average value of future poses and employing heuristic pose prediction.

Table 4 further demonstrates the robustness of the various models to changes in viewing angle and distance by showcasing the results on the synthetic test dataset of Human 3.6M. All the models were trained on the same dataset and tested on synthetic test datasets with view distance and view-angle variations. Compared to models trained on 3D joint representations, our model outperformed state-of-the-art models in both short- and long-term predictions. Compared to high-precision prediction methods, such as PGBIG and LTD, the proposed method achieved reductions of 7.2% and 10.4%, respectively, in AMPJPE. Compared with simple models, such as MMixer-3D, our model also demonstrated superior performance. To further demonstrate the performance of the proposed method, we compared it with rotation-angle-based models such as DMGNN and MMixer-Euler. The proposed method exhibits reductions of $4.0\%$ and $2.7\%$ in AMPJPE and is effective in both long- and short-term predictions.

Low-parameter models have higher training and inference speed, occupy lower computational resources, and are more interpretable. However, a simple network structure and a low parameter count may not fully capture the motion patterns, thereby reducing the robustness of the model. When the data distribution changes, the model may not maintain stable performance. As shown in Table 4, simpler models (parameter < 0.2 M) generally exhibit weaker performance than models with complex structures and a larger number of parameters. This aligns with the idea that complex structures can extract temporal and spatial information more effectively from motion sequences. MMixer-3D, which incorporates motion velocity as an input feature, outperformed other simple models, such as STSGCN and siMLPe. This is because incorporating the motion velocity reduces the reliance of the model on the joint positions. Consequently, MMixer-3D achieved a performance comparable to that of complex models such as LTD. Based on the observation that STSGCN and siMLPe have a higher AMPJPE than zero-velocity, these methods failed to output meaningful motion sequences. This indicates that the model failed to learn the intrinsic motion information and merely memorized the mapping relationship of the joint position. By encoding motion into the MS that is invariant to viewing distance and viewing angle, the proposed method achieves robust outputs with a lower parameter.

**Table 1:** Performance comparison in the first protocol between different methods via MPJPE from the Human 3.6M dataset

Millisecond	80 ms	160 ms	320 ms	400 ms	560 ms	1000 ms	Backbone
Zero-velocity [12]	19.6	32.5	55.1	64.4	-	107.9	-
LDRGCN [25]	10.7	22.5	43.1	55.8	-	97.8	GCN
Manifold-aware GAN [23]	12.6	22.5	41.9	50.8	-	96.4	GAN
Ours	**8.6**	**19.8**	**41.2**	**50.1**	-	**89.7**	MLP

The best are highlighted in bold

**Table 2:** Performance comparison in the second protocol between rotation-angle-based models via MPJPE from the Human 3.6M dataset

Millisecond	80 ms	160 ms	320 ms	400 ms	560 ms	1000 ms	Backbone	Parameter (M)
Zero-velocity [12]	24.3	45.3	78.1	90.5	109.2	137.8	-	-
DMGNN [13]	15.9	31.3	60.2	72.1	92.2	125.8	GNN	46.9
MMixer-Euler [16]	13.7	29.3	60.0	72.3	91.1	**122.8**	MLP	< 0.1
Ours	**11.3**	**26.8**	**56.7**	**69.1**	**88.8**	123.2	MLP	< 0.1

The best are highlighted in bold

**Table 3:** Performance comparison in the second protocol between simple networks method and high accuracy method via MPJPE from the Human 3.6M dataset

Millisecond	80 ms	160 ms	320 ms	400 ms	560 ms	1000 ms	Backbone	Parameter (M)
LTD [17]	12.7	26.1	52.3	63.5	81.6	114.3	GCN	2.6
Zero-velocity [12]	24.3	45.3	78.1	90.5	109.2	137.8	-	-
STSGCN [15]	17.8	34.0	57.3	68.6	85.8	117.5	GCN	< 0.1
MMixer-3D^a^ [16]	13.1	27.1	54.8	66.5	84.4	117.6	MLP	< 0.1
siMLPe^a^ [30]	10.9	24.6	53.0	65.1	84.8	119.1	MLP	0.14
PGBIG^a^ [8]	**10.6**	**23.0**	**47.8**	**58.9**	**77.3**	**110.2**	GCN	1.7
Ours	11.3	26.8	56.7	69.1	88.8	123.2	MLP	< 0.1

The best are highlighted in bold. ^a^represents the model being retrained and tested due to the use of different testing criteria

**Table 4:** Performance comparison in the second protocol between different methods via MPJPE and AMPJPE from the Human 3.6M synthetic test dataset

Millisecond	80 ms	160 ms	320 ms	400 ms	560 ms	1000 ms	AMPJPE	INV
Zero-velocity	24.4	45.6	78.6	91.1	110.0	138.6	93.4	-
siMLPe^a^	23.7	42.0	81.8	99.4	132.4	176.2	111.1	
STSGCN^a^	38.7	63.9	92.2	108.0	132.9	164.1	115.8	
MMixer-3D^a^	16.0	33.4	66.9	80.8	101.9	130.6	84.6	
LTD	16.7	33.5	65.6	79.1	100.4	133.1	84.5	
PGBIG	13.3	29.5	61.6	75.3	97.2	132.0	81.7	
DMGNN	15.9	31.4	60.4	72.3	92.4	126.2	78.9	$\surd$
MMixer-Euler	13.8	29.5	60.5	73.0	92.0	124.0	77.9	$\surd$
Ours	**11.4**	**26.9**	**57.1**	**69.6**	**89.4**	**123.9**	**75.8**	$\surd$

The best are highlighted in bold. $^{\mathrm{a}}$indicates the model with a simple network (parameter < 0.2 M). INV indicates whether the method possesses size and view angle invariance

### Results of CMU MoCap

The proposed method shows minimal prediction error at 80 ms, reducing the sense of discontinuity between observed and predicted sequences, thereby enhancing its prediction quality, as shown in Table 5. According to the results, the MPJPE of zero-velocity is greater on the CMU MoCap dataset than on the Human 3.6M dataset for long-term prediction (> 560 ms). This indicates that the long-term motions in the CMU MoCap dataset are more intense, posing a greater challenge to long-term prediction. Compared to the manifold-aware GAN in the first protocol, the proposed method achieves state-of-the-art results at 80 and 160 ms.

**Table 5:** Performance comparison in the first protocol between different methods via MPJPE from the CMU MoCap dataset

Millisecond	80 ms	160 ms	320 ms	400 ms	560 ms	1000 ms
Zero-velocity [12]	18.4	31.4	56.2	67.7	-	130.5
LDRGCN [25]	9.4	17.6	31.6	43.1	-	82.9
Manifold-aware GAN [23]	9.4	15.9	**29.2**	**38.3**	-	**80.6**
Ours	**5.4**	**13.8**	31.5	39.8	-	85.0

The best are highlighted in bold

**Table 6:** Performance comparison between different methods via MPJPE and AMPJPE from the CMU Mocap synthetic test dataset

Millisecond	80 ms	160 ms	320 ms	400 ms	560 ms	1000 ms	AMPJPE
Zero-velocity	19.7	38.1	71.0	85.5	109.8	149.5	74.7
MSR-GCN	14.2	30.2	66.4	85.1	119.7	183.1	92.1
PGBIG	10.4	23.2	54.1	71.8	107.0	180.7	85.0
LTD	16.7	32.9	63.9	78.4	104.6	149.3	72.1
DMGNN	15.4	29.1	52.6	62.4	76.9	103.6	69.3
Ours	**6.5**	**16.4**	**37.7**	**47.8**	**65.9**	**101.8**	**45.9**

The best are highlighted in bold

Table 6 presents the testing results for a synthetic dataset that includes viewing angle and distance variations. Owing to the viewing distance and viewing angle invariance of the proposed method, the performance of the synthetic dataset did not deteriorate compared with that of the standard dataset. Compared with DMGNN, the proposed method reduced MPJPE by $1.7\%$ at 1000 ms and AMPJPE by 33.8$\%$. The results demonstrate that the proposed method has a significant advantage in terms of short-term predictions. In addition, based on the results, the complex models PGBIG and LTD exhibited a more significant performance degradation on the synthetic dataset of CMU MoCap than on Human 3.6M. This is because the CMU MoCap dataset contains fewer training samples and more intense motions for long-term prediction, leading to a decrease in the baseline performance.

### Time comparisons

Table 7 lists the comparative run times of the various methods. During both the training and inference stages of the network, the proposed method, which uses a simpler structure and lower parameter count, has lower training and inference times. However, the proposed method requires additional computational time during both encoding and decoding stages. Considering a single data input scenario in the application, the encoding and decoding time for each motion is 13 ms, which is significantly smaller than the minimum time requirement for motion prediction (80 ms), and can still meet the requirements of the application.

**Table 7:** Comparison of training time, inference time, and encoding time

Method	Train (per batch)	Test (per batch)	Encoding-decoding (per sample)
DMGNN	473 ms	85 ms	-
LTD	114 ms	30 ms	-
PGBIG	145 ms	43 ms	-
Ours	**51 ms**	**26 ms**	13 ms

The best are highlighted in bold

### Visualization results

**Fig. 10:**
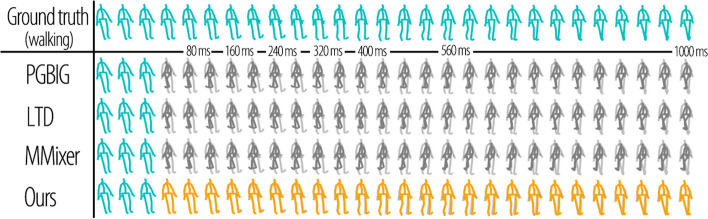
Visualization results in comparison on walking of Human 3.6M synthetic dataset with size and rotation noise. The first row shows the ground truth, and the following rows are the results of PGBIG, LTD, MMixer, and ours

**Fig. 11:**
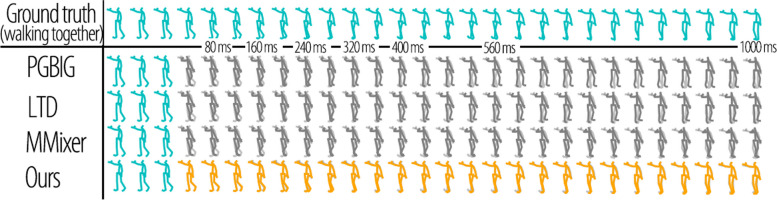
Visualization results in comparison on walking-together of Human 3.6M synthetic dataset with size and rotation noise. The first row shows the ground truth, and the following rows are the results of PGBIG, LTD, MMixer, and ours

To provide a clearer illustration of the advantages of the proposed method, we visualized the predicted results for multiple actions in the Human 3.6M dataset in Figs. 10 and 11. These figures demonstrate the effectiveness of our method in handling the invariance of viewing distance and viewing angles effectively. In the figures, cyan represents the input sequences, light grey represents the ground truth, dark grey represents the results of the previous methods, and orange represents the predictions of the proposed method. The proposed method effectively addresses changes in the viewing distance and angle of the input sequence. The previous methods could not accurately predict the perspective and size of the skeleton, as shown by the predictions at 40 ms in Figs. 10 and 11. Because of the lack of invariance in these methods, they can only convert motions into a standard representation through preprocessing to predict human motions in a scene. Subsequently, the predicted sequences were converted back to the original scene through postprocessing. Unlike our proposed method, which directly addresses changes in view distance and angle to deliver more accurate and reliable predictions, the existing approach proves to be less effective.

Overall, the visualized results further demonstrate the superior performance and robustness of our proposed method in handling variations in viewing distance and viewing angle during motion prediction tasks.

### Ablation analysis

#### Performance on various test datasets

Table 8 presents the AMPJPE for the various test datasets. The performance of the proposed method was unaffected by variations in the viewing distance and viewing angle. The proposed method achieves the best performance on test datasets with viewing angle variations and datasets that encompass variations in both the viewing distance and viewing angle. The loss function of the proposed method emphasizes motion evolution rather than joint position. The loss function has greater potential than the loss function based on the joint position to predict motion in diverse postures, such as walking. The proposed loss function sacrifices performance to achieve robustness against postural variations. The outputs are slightly different from the ground truth in the long term, but they were still reasonable in the proposed method. Consequently, its performance on standard datasets is inferior to that of the methods that employ MPJPE as the loss function. However, the proposed method remains the most effective model with a simple network when it originates to variations in view distance and view angle. The performance degradation of the simpler models was more severe than that of the larger models. Experimental results showed that models such as STSGCN and siMLPe struggle to extract meaningful motion information. Consequently, their performance significantly declined when the test data deviated from the training data distribution.

**Table 8:** Performance comparison between different methods using AMPJPE on various Human 3.6M

Method	Standard	View-distance	View-angle	Both	Parameter(M)
Zero-velocity	92.80	93.42	92.80	93.42	0
LTD	69.21	72.72	82.90	84.50	2.6
PGBIG	**66.27**	**70.07**	80.17	81.65	1.7
DMGNN$^{\mathrm{a}}$	78.63	78.89	78.63	78.89	46.9
STSGCN	75.43	88.70	103.92	115.80	$< 0.10$
MMixer-Euler$^{\mathrm{a}}$	77.14	77.89	77.14	77.89	$< 0.10$
MMixer-3D	72.19	75.02	83.28	84.61	$< 0.10$
siMLPe	71.91	96.72	88.18	111.09	0.14
Ours	75.40	75.77	**75.40**	**75.77**	$< 0.10$

The best are highlighted in bold. $^{\mathrm{a}}$indicates a method that is based on 3D joint rotational angles. Standard: unprocessed dataset; View-distance: skeleton template scaling; View-angle: skeleton template rotation; Both: skeleton template scaling and rotation

**Table 9:** Comparison of AMPJPE on different encoding spaces for the Human 3.6M dataset

	Coding space	Standard	Scale	View-angle	Both
(1)	Latent spaces of PCA	86.50	93.10	151.13	155.71
(2)	3D joint coordinate	79.62	85.83	117.42	119.37
(3)	pre-MS with geodesic loss	79.43	79.93	116.76	117.51
(4)	MS	**75.40**	**75.77**	**75.40**	**75.77**

The best are highlighted in bold

#### Motion coding

We constructed a latent space using PCA to replace the proposed MS, and demonstrated the advantages of the novel encoding space. As a latent space constructed based on PCA cannot be proven to be isometric to the original space, optimization in this space cannot achieve the optimal solution in the 3D joint coordinates. Table 9 (1) shows that the space constructed using the PCA method does not effectively predict motion.

We designed a contrasting experiment to demonstrate the efficiency of geometric coding, as shown in Table 9. The AMPJPE of the proposed method decreased by 5.3% compared with 3D joint coordinates using Euclidean loss and decreased by 5.1% compared with pre-MS using geodesic loss on the standard test dataset. There was no significant difference in the performance between (3) and (2) on the standard test dataset. This is because geodesic distance is an extension of the Euclidean distance on the manifold. The performance improvement in coding motions into the MS stems from the fact that the distance based on motion evolution can effectively measure the distance between different motion patterns. By comparing the performance on the scale test dataset, it was observed that pre-MS successfully eliminated the equivalence classes associated with the view distance. The results on the view-angle dataset for (4) and (3) demonstrate that the models trained on the MS are unaffected by view-angle changes. The results presented in Table 9 demonstrate that the proposed method effectively addresses the challenges posed by variable viewing distances and viewing angles in practice. The adopted geometric coding method provides valuable insights for the future.

**Table 10:** Influence of $\gamma$ c (AMPJPE)

Millisecond	80 ms	160 ms	320 ms	400 ms	560 ms	1000 ms
$\gamma =$ 0	12.88	29.45	60.03	72.46	92.21	125.79
$\gamma =$ 0.5	12.10	28.18	58.71	71.23	91.16	125.68
$\gamma =$ 1	11.36	**26.91**	**57.05**	**69.56**	**89.43**	**123.88**
$\gamma =$ 2	**11.26**	26.92	57.81	70.84	91.84	128.45

The best are highlighted in bold

#### Encoding distribution

**Fig. 12:**
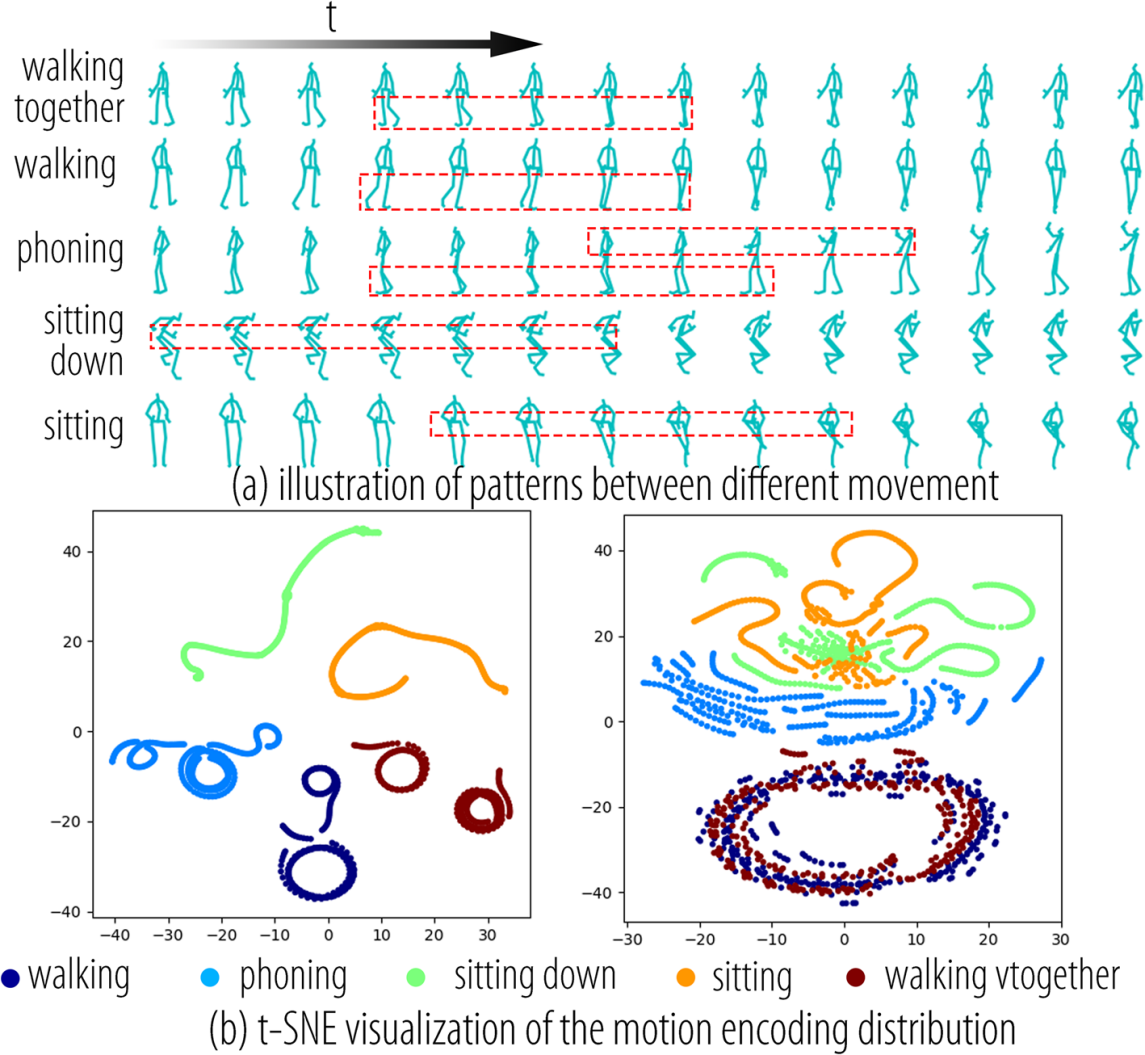
t-SNE visualization of the distribution of motion representation for the five motion classes in the Human 3.6 dataset. **a** The patterns between different movements; **b** t-SNE visualization of the motion encoding distribution, 3D joint coordinates, and SP-TSRVF encoding space

A characteristic of prediction tasks is that the input observation sequences are not necessarily similar in motion, even if they have the same label. Some input sequences such as transitional motions may not be directly related to their corresponding labels. Therefore, for prediction tasks, the distribution of data represented in 3D joint coordinates is no longer concentrated, but rather exhibits a strip-like distribution, as shown on the left side of Fig. 12b. The observations showed that motion sequences with different labels, such as walking and walking, exhibited the same motion patterns. Figure 12a shows that the motion patterns of walking and walking together are the same and involve cyclic leg movements. The motion patterns of sitting and sitting down were similar, involving movements of the torso and upper limbs but with differences in leg details. We aimed to construct an encoding space in which the distribution of motion sequences with the same pattern was as similar as possible. As shown on the right side of Fig. 12b, encoding based on MS exhibits an ideal distribution in which the data distribution of motion sequences with the same pattern, such as walking together and walking, is consistent. The distributions of sitting and sitting down were close to each other but still exhibited some differences. Because phoning involves both upper-body and leg movements, it falls between sitting and walking with regard to motion patterns. The encoding method based on SP-TSRVF successfully extracted the motion patterns. This reduces the demand for expressive capacity and network complexity.

#### Balanced coefficient

Table 10 presents a comprehensive exploration of the balanced coefficient $\gamma$. Setting this parameter balanced the accumulated error during curve integration. The network was encouraged to focus on the early stages by assigning a higher weight to short-term predictions. This approach aims to reduce the prediction errors in the initial stages and improve the prediction performance. Experiments demonstrated that the best performance was achieved when $\gamma =1$. At 1000 ms, applying $\gamma =0$ resulted in a 1.5% reduction in MPJPE compared with not applying any weighting ($\gamma =0$). The performance improvement was particularly notable in short-term predictions, with an 11% reduction observed at 80 ms for MPJPE. However, as $\gamma$ increases, over-emphasizing short-term predictions can cause the network to completely neglect medium- and long-term predictions. When $\gamma =2$, there was a slight reduction of 0.8% over $\gamma =1$ at 80 ms in MPJPE. However, this advantage quickly diminishes, and the performance of both approaches becomes comparable at 160 ms. $\gamma =2$ exhibits a -3.7% performance change at 1000 ms from $\gamma =1$.

**Table 11:** Influence of different parts of the SF block on MPJPE and AMPJPE for the Human 3.6M dataset

Millisecond	80 ms	160 ms	320 ms	400 ms	1000 ms	AMPJPE
w/o F-MLP	12.91	29.66	60.95	73.85	128.99	79.80
w/o S-MLP	12.86	31.07	65.36	79.02	133.14	83.44
Ours	**11.36**	**26.91**	**57.05**	**69.56**	**123.88**	**75.77**

The best are highlighted in bold

**Table 12:** Influence of the SF block number on AMPJPE for the Human 3.6M dataset

SF block number	4	8	10	12	16
AMPJPE	78.28	77.42	**75.77**	76.67	76.48

The best are highlighted in bold

#### SF block

The SF block is the most crucial component of the network. This module consists of the S-MLP and F-MLP, which are responsible for establishing relationships between bones and motion components. As shown in Table 11, both S-MLP and F-MLP play important roles in prediction. By removing the F-MLP or S-MLP, the AMPJPE increased by 5.4% and 10.1% at 1000 ms, respectively. S-MLP significantly affects performance improvement. This is because the S-MLP can establish correlations between different bones, such as by coordinating the movements of the hands and legs during walking. The number of SF blocks is important for motion prediction. An insufficient number of SF blocks may fail to capture adequate motion information, while an excessive number can result in overfitting, diminishing the generalizability of the model. Table 12 presents the experimental results, which indicate that the optimal prediction performance is achieved when there are 10 SF blocks.

## Conclusions

This study proposes a novel framework that enhances the generalization of motion prediction models using geometric encoding. The proposed framework enables the prediction of motions with arbitrary view distances and angles, thereby significantly expanding the applicability of the model. Experimental results on the Human 3.6M and CMU MoCap datasets demonstrate that the proposed method successfully manages viewing distance and viewing angle variations. The ablation experiments demonstrate that there are diverse invariances across the different spaces constructed, such as view distance invariance in pre-MS, and both view distance and view angle invariance in MS. t-SNE visualization shows that the proposed MS effectively encodes motion patterns, enabling motion modeling with simple models (e.g., MLPs). The proposed encoding method applies to motion prediction and holds potential for applications in motion recognition, motion segmentation, and other related fields.

## Data Availability

The Human 3.6M dataset can be obtained from an official website (https://vision.imar.ro/human3.6m). The datasets generated and analyzed in the current study can be accessed at Zenodo (https://doi.org/10.5281/zenodo.10716924). The data include geometrically encoded data and predictive results.

## Abbreviations

PS: Posture space
Pre-MS: Pre-motion space
MS: Motion space
SRVF: Square-root velocity function
TSRVF: Transport square-root velocity function
SP-TSRVF: Specified path transport square-root velocity function
MPJPE: Mean per joint position error
AMPJPE: The average MPJPE for 0-1000 ms
S-MLP: Spatial MLP
F-MLP: Frequency MLP
ST: Spatial-temporal
RNN: Recurrent neural network
CNN: Convolutional neural network
GCN: Graph convolutional network
MLP: Multi-layer perceptron
DCT: Discrete cosine transform
GAN: Generative adversarial network
3D: Three-dimensional
SF: Spatial-frequency
